# Extreme Reconfiguration of Plastid Genomes in Papaveraceae: Rearrangements, Gene Loss, Pseudogenization, IR Expansion, and Repeats

**DOI:** 10.3390/ijms25042278

**Published:** 2024-02-14

**Authors:** Jialiang Cao, Hongwei Wang, Yanan Cao, Shenglong Kan, Jiamei Li, Yanyan Liu

**Affiliations:** 1 College of Plant Protection, Henan Agricultural University, Zhengzhou 450002, China; caojlcao@163.com (J.C.); whwcas@163.com (H.W.); caoyn47@163.com (Y.C.); 2 Marine College, Shandong University, Weihai 264209, China; kanshenglong@sdu.edu.cn; 3 College of Life Sciences, Henan Agricultural University, Zhengzhou 450046, China

**Keywords:** Papaveraceae, plastomes, rearrangement, IR expansion, gene loss

## Abstract

The plastid genomes (plastomes) of angiosperms are typically highly conserved, with extreme reconfiguration being uncommon, although reports of such events have emerged in some lineages. In this study, we conducted a comprehensive comparison of the complete plastomes from twenty-two species, covering seventeen genera from three subfamilies (Fumarioideae, Hypecooideae, and Papaveroideae) of Papaveraceae. Our results revealed a high level of variability in the plastid genome size of Papaveraceae, ranging from 151,864 bp to 219,144 bp in length, which might be triggered by the expansion of the IR region and a large number of repeat sequences. Moreover, we detected numerous large-scale rearrangements, primarily occurring in the plastomes of Fumarioideae and Hypecooideae. Frequent gene loss or pseudogenization were also observed for *ndhs*, *accD*, *clpP*, *infA*, *rpl2*, *rpl20*, *rpl32*, *rps16*, and several tRNA genes, particularly in Fumarioideae and Hypecooideae, which might be associated with the structural variation in their plastomes. Furthermore, we found that the plastomes of Fumarioideae exhibited a higher GC content and more repeat sequences than those of Papaveroideae. Our results showed that Papaveroideae generally displayed a relatively conserved plastome, with the exception of *Eomecon chionantha*, while Fumarioideae and Hypecooideae typically harbored highly reconfigurable plastomes, showing high variability in the genome size, gene content, and gene order. This study provides insights into the plastome evolution of Papaveraceae and may contribute to the development of effective molecular markers.

## 1. Introduction

The plastid genome (plastome) has quickly become a commonly used marker in plant systematic phylogenetic research due to its advantages of structural conservation, small genome size (approximately 150–200 Kb), and uniparentally inheritance [1,2,3]. The plastomes of higher plants have a highly conserved quadripartite circular structure, comprising a large single-copy region (LSC) and a small single-copy region (SSC) separated by two inverted repeat regions (IR) [4]. Although some atypical forms such as the extreme contraction of IRs, entire IR loss, and direct repeats (DRs) have been detected, the typical plastome architecture, including genome size, gene content, and gene order, is generally highly conserved, with two configurations usually coexisting and interchanging via IRs [5]. Nevertheless, an increasing number of studies have revealed a structural variation, including large-scale rearrangements in Fabaceae [6,7,8,9,10], Geraniaceae [11,12,13,14], Cactaceae [15,16], Campanulaceae [17,18], and Passifloraceae [19,20,21,22], as well as large IR contractions in Schisandraceae [23] and Lauraceae [24], and even loss of one IR region in Leguminosae [7,25], Geraniaceae [12], and Passifloraceae [20].

Papaveraceae, one of the most diverse families of Ranunculales, consists of about 850 species belonging to 45 genera and predominantly occur in forests, subalpine meadows, alpine tundra, grasslands, and deserts, especially in temperate regions of the northern hemisphere [26]. This family exhibits a high degree of endemism, with over 70% of its species being geographically restricted [26]. Papaveraceae is renowned for the opium poppy (*Papaver somniferum*), which can serve as a source of analgesic drugs [27]. In addition, *Corydalis yanhusuo*, *Dactylicapnos torulosa*, *Chelidonium majus*, *Macleaya cordata*, *Hylomecon japonica*, *Hypecoum erectum*, and *Eomecon chionantha* are also used in traditional medicine [28,29,30,31,32,33,34]. The taxonomy and phylogeny of Papaveraceae have been a subject of ongoing debate. Hoot et al. [35] divided Papaveraceae into three subfamilies, i.e., Pteridophylloideae, Papaveroideae, and Fumarioideae. Wang et al. [36] divided Papaveraceae into two subfamilies, i.e., Fumarioideae (including *Pteridophyllum* and *Hypecoum*) and Papaveroideae. Afterwards, Hoot et al. [37] divided Papaveraceae into four subfamilies, i.e., Pteridophylloideae, Papaveroideae, Hypecoideae, and Fumarioideae, and Pteridophylloideae was supported as a sister to the other subfamilies and Hypecoideae as a sister to Fumarioideae. Although there is still controversy about the systematic position of the two single-genera subfamilies Hypecooideae and Pteridophylloideae, this classification is currently generally accepted [26].

Within Fumarioideae, Park et al. [38] firstly reported the large-scale rearrangements of plastomes in *Lamprocapnos* and speculated that the expansion and contraction of the IR region might contribute to these rearrangements. Additionally, Xu and Wang revealed *Corydalis* to be another unusual lineage with extensive large-scale plastome rearrangements and significant pseudogenizations or losses of the *accD*, *clpP,* and *ndh* genes [39]. Furthermore, recent wide-scale comparative studies confirmed the extensive rearrangements and gene losses throughout the plastomes of *Corydalis* [40,41]. Moreover, the plastomes of *Fumaria* also showed signatures of structural rearrangement and loss of the *accD* gene [41]. In contrast, the plastomes of Papaveroideae appeared more conserved, with no structure rearrangements and gene losses detected, despite the release of plastomes from several genera [42]. For other closely related Ranunculales taxa, Sun et al. [43] reported plastid rearrangements of Circaeasteraceae, and found an inversion of approximately 49 kb and 3.5 kb in the LSC region, with pseudogenizations or losses of the *accD* and *ndh* genes. Nevertheless, the plastomes of Eupteleaceae, Lardizabalaceae, Menispermaceae, Berberidaceae, and Ranunculaceae were generally conserved [44,45], with the exception of several genera, such as in the cases of the inversion of a 44.8 kb segment in the LSC region and loss of the *rpl32* gene in *Adonis* [46], and the small expansion of the IR region in *Mahonia* and *Asteropyrum* [47,48].

Structural variations are fundamental characteristics of a specific group [49]. Therefore, inferring structural variations of the plastomes in diverse phylogenetic lineages is an interesting research topic, and the results may provide more insights into their evolutionary history. Despite the recent surge in complete plastome sequencing, the comprehensive comparison has been largely confined to a few genera in Fumarioideae, such as *Corydalis*, *Lamprocapnos,* and *Fumaria* [38,39,40,41], resulting in relatively little knowledge about genomic changes throughout Papaveraceae. In this study, we carried out a detailed comprehensive comparative analysis for the plastomes of Papaveraceae with expanded sampling, aiming to characterize the plastomes from major lineages of Papaveraceae and investigate the factors contributing to plastome variation. Our study will advance our understanding of the plastome diversity and evolution in plants.

## 2. Results

### 2.1. Chloroplast Genome Characterization

**Figure 1:**
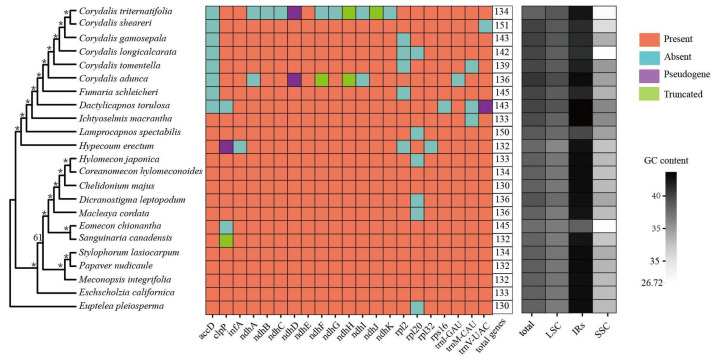
Gene loss and GC content of the Papaveraceae plastomes. The left tree was constructed for twenty-three taxa based on ninety-one common unique plastid genes with maximum likelihood (ML) analyses. Asterisks (*) represent 100% bootstrap value.

A total of 185 Gb 150 bp PE Illumina reads were generated. After quality control using SOAPnuke, approximately an average of 7.2 GB of clean data were generated for each sample (Table S1). The plastomes of Papaveraceae exhibited a standard cyclic quadripartite structure comprising two single-copy regions (LSC and SSC), separated by a pair of inverted repeats (IRa and IRb). The plastid genome size differed significantly, ranging from 151,864 bp (*Meconopsis integrifolia*) to 219,144 bp (*Corydalis sheareri*). The length of LSC ranged from 76,421 bp (*Dactylicapnos torulosa*) to 100,365 bp (*Corydalis adunca*), the length of SSC varied from 136 bp (*Eomecon chionantha*) to 31,701 bp (*Dactylicapnos torulosa*), and the IR lengths ranged from 25,653 bp (*Meconopsis integrifolia*) to 62,384 bp (*Corydalis sheareri*) (Table 1). The overall GC content of Papaveraceae varied from 37.9% (*Eomecon chionantha*) to 41.1% (*Corydalis adunca*), with the lowest GC content observed in the SSC region and a relatively higher content in the IR region. Notably, *Corydalis*, *Lamprocapnos*, and *Eomecon* exhibited a disproportionately lower GC content (Figure 1).

The number of protein-coding genes (PCGs) ranged from 85 (*Corydalis triternatifolia*) to 100 (*Lamprocapnos spectabilis*), and tRNAs varied from 36 (*Corydalis adunca*) to 44 (*Corydalis sheareri*) (Figure 1, Table 1). All Papaveraceae species shared 91 unique plastid genes, including 60 PCGs, 27 tRNA genes, and 4 rRNA genes. Of those, ten genes harbored more than one intron (*atpF*, *petB*, *petD*, *rpl16*, *rpoC1*, *rps12*, *ycf3*, *trnA-UGC*, *trnK-UUU*, *trnL-UAA*), and the largest intron was observed in *trnK-UUU* (~2400 bp), which contains the *matK* gene. In addition, ten PCGs (*ccsA*, *matK*, *psaC*, *psaI*, *psbA*, *rpl22*, *rpl23*, *rps15*, *rps3*, *ycf1*), four rRNAs (*rrn16*, *rrn23*, *rrn4.5*, *rrn5*), and eight tRNAs (*trnH-GUG*, *trnK-UUU*, *trnQ-UUG*, *trnI-CAU*, *trnL-UAG*, *trnT-UGU*, *trnG-GCC*, *trnfM-CAU*) were duplicated in the IR regions of some species. Gene losses occurred frequently in Papaveraceae, especially in the subfamily Fumarioideae. For example, the *accD* gene was detected as lost in *C. triternatifolia*, *C. gamosepala*, *C. tomentella*, *C. sheareri*, *C. longicalcarata*, *C. adunca*, *Dactylicapnos torulosa,* and *Fumaria schleicheri*. The *clpP* gene was lost in *Dactylicapnos torulosa* and *Eomecon chionantha*. The *ndhA* and *ndhI* genes were lost in *Corydalis triternatifolia* and *C. adunca*. The *ndhB*, *ndhC, ndhF*, *ndhG,* and *ndhK* genes were lost in *C. triternatifolia*. The *trnV-UAC* gene was lost in *C. sheareri*. Additionally, three genes (*ndhF*, *ndhH,* and *ndhJ*) were truncated in two species of *Corydalis*, and three genes (*clpP*, *ndhD*, *trnV-UAC*) were identified as pseudogenes in *Corydalis* or *Dactylicapnos*. In sharp contrast with the frequent gene loss or pseudogenization in the subfamily Fumarioideae, only *clpP* was observed as pseudogenization, lost, or truncated in three species (*Hypecoum erectum*, *Eomecon chionantha,* and *Sanguinaria canadensis*) of Papaveroideae and Hypecooideae (Figure 1).

### 2.2. Phylogenetic Analyses

The maximum likelihood (ML) tree was constructed based on the 91 shared genes. The aligned length of the concatenated plastid genes was 76,127 bp, with 12,340 variable sites (22.42%) and 6449 parsimony informative sites (12.08%) (gaps were not included). Our robust phylogeny recovered a largely congruent topology with previous studies [26]. As expected, three subfamilies were recovered in the high-confidence phylogeny, and Papaveroideae was strongly supported as a sister to a solid monophyletic group comprising all species from Hypecooideae and Fumarioideae (Figure 1A and Figure S1). Within Papaveroideae, Chelidonieae and Papavereae were recovered as non-monophyly due to the exceptional position of *Stylophorum lasiocarpum*. Additionally, the relationships of Chelidonieae, Papavereae, and Eschscholzieae have not been fully resolved. Within Fumarioideae, Fumarieae was nested in Corydaleae and sister to *Corydalis*.

### 2.3. Genome Structure Variations

**Figure 2:**
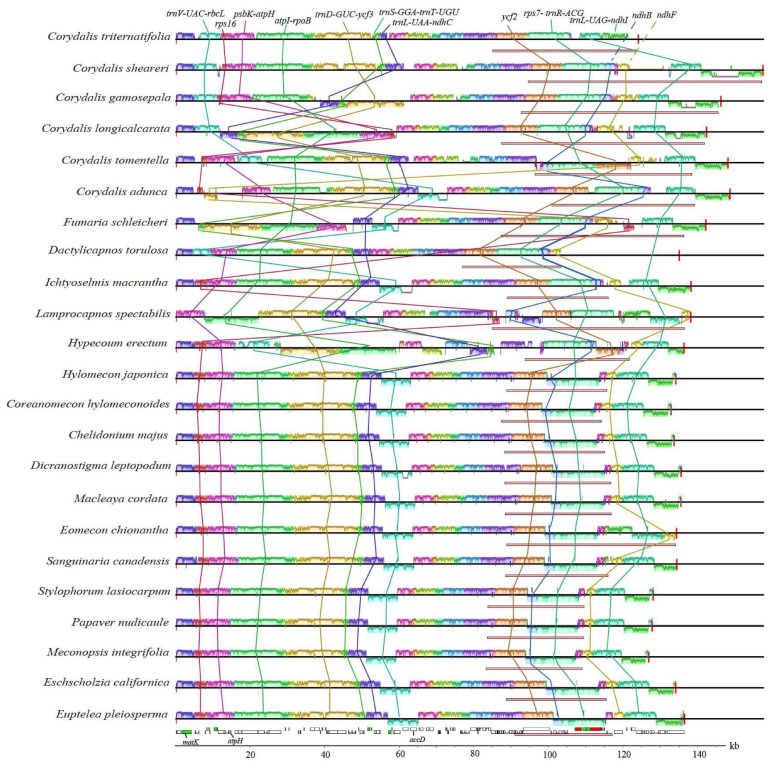
Structural alignments of Papaveraceae plastomes using Mauve with Euptelea pleiosperma as reference. Colorful blocks represent locally collinear blocks (LCBs), and lines connecting the blocks indicate homology. Only one copy of the inverted repeat (IR) is shown and the pink boxes below the plastome indicates its IR region.

Multiple alignment analysis across twenty-three species showed the presence of several locally collinear blocks, suggesting that the plastomes of Papaveraceae might have undergone varying degrees of rearrangement (Figure 2). Firstly, one block (~6 kb) containing five genes (*trnV-UAC*, *trnM-CAU*, *atpE*, *atpB*, and *rbcL*) and the associated non-coding sequences relocated from the typically posterior part of the LSC region to the front. This block was relocated downstream of the *matK* gene in *Corydalis triternatifolia*, *C. sheareri*, *C. gamosepala*, *C. longicalcarata,* and *Dactylicapnos torulosa*. In *Corydalis tomentella* and *Hypecoum erectum*, this block was relocated downstream of the *atpH* and *accD* genes, respectively. In addition, these five genes in *Corydalis triternatifolia*, *C. sheareri*, *C. gamosepala*, *C. longicalcarata*, *C. tomentella*, *Dactylicapnos torulosa,* and *Hypecoum erectum* were inverted, a phenomenon not observed in Papaveroideae. The remaining species exhibited a typical position of angiosperms downstream of the *ndhC* gene. Secondly, the *rps16* (~1 kb) gene was relocated from the typically front part of the LSC region to the posterior in *Corydalis longicalcarata*. In *Fumaria schleicheri* and *Lamprocapnos spectabilis*, the *rps16* gene transferred from the typically front part of the LSC region to the IR region. Furthermore, the *rps16* gene was inverted in *Corydalis longicalcarata*, *Fumaria schleicheri,* and *Lamprocapnos spectabilis*. Thirdly, one block (~7 kb) containing eight genes (*psbK*, *psbI*, *trnS*-*GCU*, *trnG-UCC*, *trnR-UCU*, *atpA*, *atpF,* and *atpH*) relocated from the typically front part of the LSC region to the posterior in *Corydalis longicalcarata*. Moreover, this block was inverted in *Corydalis longicalcarata* and *Fumaria schleicheri*. Fourthly, one block (~14–15 kb) comprising five genes (*atpI*, *rps2*, *rpoC2*, *rpoC1*, and *rpoB*) relocated from the typically front part of the LSC region to the posterior in *Hypecoum erectum*. In addition, in *Corydalis longicalcarata*, *Fumaria schleicheri*, *Lamprocapnos spectabilis,* and *Hypecoum erectum*, this block was inverted. Fifthly, one block (~13–15 kb) containing fourteen genes (from *trnD-GUC* to *ycf3*) was inverted in *Corydalis gamosepala*, *C. longicalcarata*, *Fumaria schleicheri*, and *Hypecoum erectum*. And in *Corydalis longicalcarata* and *Fumaria schleicheri*, it was relocated from the middle to the front of the LSC region. Sixthly, one block (~2 kb) containing three genes (*trnS-GGA*, *rps4,* and *trnT-UGU*) relocated from the typically middle of the LSC region to the posterior in *Hypecoum erectum*, while it was relocated from the typically middle of the LSC region to the front in *Corydalis longicalcarata* and *Fumaria schleicheri*. And in *Corydalis gamosepala*, *C. longicalcarata*, *Fumaria schleicheri*, and *Hypecoum erectum*, this block was inverted. Seventhly, one block (~2 kb) containing five genes (*trnL-UAA, trnF-GAA, ndhJ, ndhK,* and *ndhC*) was inverted in *Hypecoum erectum*, *Corydalis longicalcarata,* and *C. gamosepala*. Eighthly, one block (~7 kb) in the IR region containing one gene (*ycf2*) relocated from the typically front of the IR region to the posterior in *Hypecoum erectum*. And this block was inverted in *Corydalis tomentella* and *Hypecoum erectum*. Ninthly, one block (~7 kb) in the IR region containing the *ndhB* gene was inverted in all taxa of Fumarioideae and Hypecooideae except for *Corydalis tomentella*. Similarly, one block (~11 kb) in the IR region containing ten genes (from *rps7* to *trnR-ACG*) was inverted in all sampled species of Fumarioideae and Hypecooideae except for *C. tomentella*. Tenthly, one block (~2 kb) in the SSC region only containing the *ndhF* gene relocated to the IR region in *Eomecon chionantha, Dactylicapnos torulosa*, *Fumaria schleicheri*, *Corydalis tomentella, C. longicalcarata, C. gamosepala,* and *C. sheareri* due to the expansion of the IR region. However, this block was relocated to the LSC region in *C. adunca*. In addition, in *Eomecon chionantha* and *Corydalis adunca,* it was inverted. Lastly, one block (~6 kb) in the SSC region, which included seven genes (from *trnL-UAG* to *ndhI*), relocated to the IR region in *Eomecon chionantha, Fumaria schleicheri*, *Lamprocapnos spectabilis,* and all six *Corydalis* species due to the expansion of the IR region absorbing the SSC region, and was inverted in *Lamprocapnos spectabilis* and *Eomecon chionantha*. In conclusion, we observed that relocations and inversions were widely distributed in Papaveraceae, especially in Hypecooideae and Fumarioideae. Within Papaveroideae, the rearrangement was mainly concentrated in some specific lineage, such as *Eomecon* (Figure 2).

**Figure 3:**
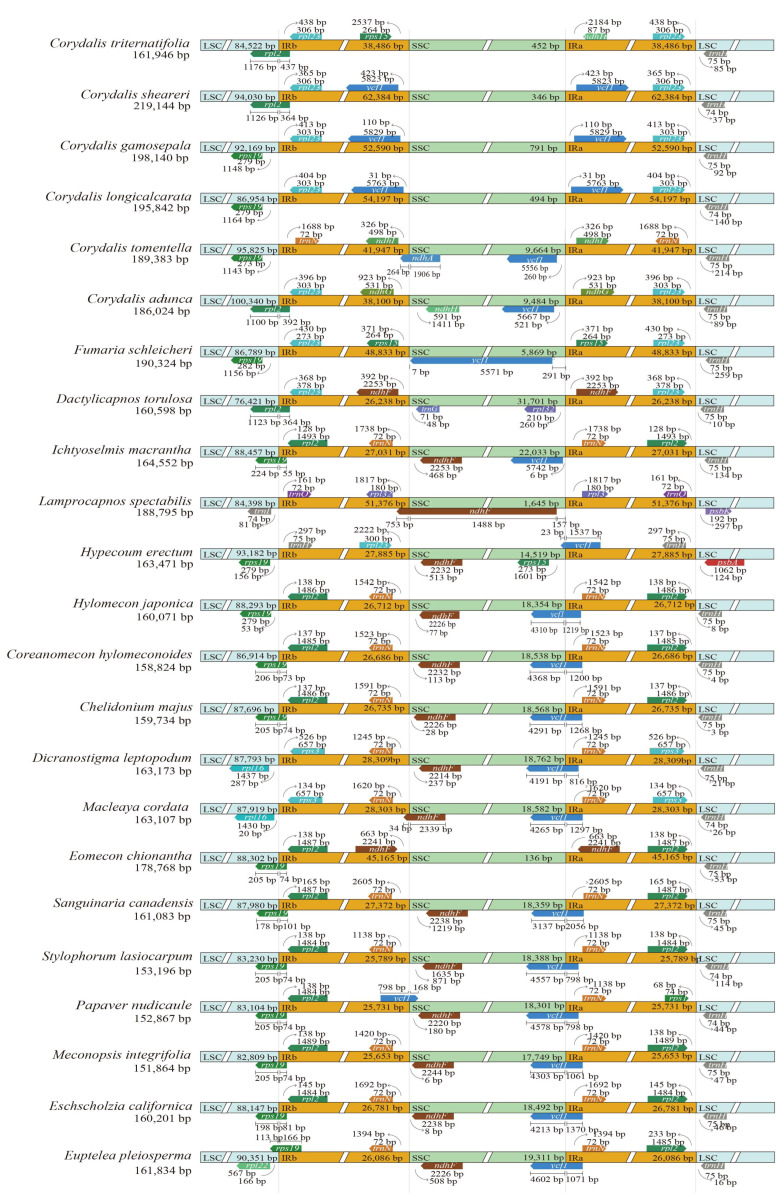
Comparison of the borders of LSC, SSC, and IR regions among Papaveraceae plastomes. The distance in the figure is not to scale.

The IR boundary analyses indicated that the IR regions of Papaveroideae plastomes were highly conserved, while the IR boundaries of Hypecooideae and Fumarioideae genomes exhibited high variation (Figure 3). In Papaveroideae, most species had similar structures: the *rps19* gene was located in the LSC/IRb boundary, the intergenic region between the *trnN* and *ndhF* genes resided precisely at the IRb/SSC boundary, the *ycf1* gene crossed the IRa/SSC boundary, and the *trnH* gene was located in the IRa/LSC boundary. Specifically, in *Papaver nudicaule*, the *ycf1* gene resided precisely at the IRb/SSC boundary. In *Eomecon chionantha*, the IRb region extended into the SSC region, absorbing the *ndhF* gene. Whereas, in *Macleaya cordata* and *Dicranostigma leptopodum*, the IR region expanded into the LSC region, assimilating the *rps19, rpl22,* and *rps3* genes, thereby establishing the *rps3* gene’s placement at the IR/LSC boundary. In the subfamily Fumarioideae and Hypecooideae, the *rps19* gene was located in the LSC region in *Corydalis gamosepala*, *C. longicalcarata*, *C. tomentella*, *Fumaria schleicheri,* and *Hypecoum erectum* at distances ranging from 156 bp to 1156 bp from the LSC/IRb boundary, while the *rps19* gene resided precisely at the LSC/IRb boundary in *Ichtyoselmis macrantha*. The *rpl2* gene was located in the LSC/IRb boundary in *Corydalis triternatifolia, C. sheareri, C. adunca,* and *Dactylicapnos torulosa*, while in *Lamprocapnos spectabilis,* the LSC/IRb boundary was situated between the *trnI* and *trnQ* genes. For the IRb/SSC boundary of Fumarioideae and Hypecooideae, the *ycf1* gene was located within the IRb region in *Corydalis sheareri, C. gamosepala,* and *C. longicalcarata*, with a distance of 31 bp to 423 bp from the boundary. Furthermore, the *ndhF* gene was located near the IRb/SSC boundary in *Dactylicapnos torulosa, Ichtyoselmis macrantha, Lamprocapnos spectabilis,* and *Hypecoum erectum*. The *rps15* gene was located near the IRb/SSC boundary in *Corydalis triternatifolia* and *Fumaria schleicheri*, and in *C. adunca,* the IRb/SSC boundary was located between the *ndhG* and *ndhH* genes. Nevertheless, in *C. tomentella*, the *ndhA* gene was located at the IRb/SSC boundary. For the IRb/SSC boundary of Fumarioideae and Hypecooideae, the *ycf1* gene was located near the boundary in *Corydalis sheareri*, *C. gamosepala*, *C. longicalcarata*, *C. tomentella*, *C. adunca*, *Fumaria schleicheri,* and *Ichtyoselmis macrantha*, with a distance of 6 bp to 521 bp from the boundary, and the *ycf1* gene of *Hypecoum erectum* was exactly located at the boundary. In *Corydalis triternatifolia*, the *ndhH* gene was 2184 bp away from the IRb/SSC boundary. In *Dactylicapnos torulosa*, the IRb/SSC boundary was situated between the *rpl32* and *ndhF* genes, and in *Lamprocapnos spectabilis*, the *ndhF* gene was 157 bp away from the boundary. For the IRa/SSC boundary of Fumarioideae and Hypecooideae, the *trnH* gene was located at a distance of 10 bp to 297 bp from the boundary in all sampled species with the exception of *L. spectabilis*, in which the boundary was situated between the *trnQ* and *psbK* genes.

### 2.4. Codon Usage and Repeat Sequence Analysis

Codon with RSCU values greater than one was considered to have relatively high usage frequencies. We examined the codon usage frequency of PCGs in Papaveraceae and found that eighteen codons encoding eighteen amino acids had RSCU values > 1 (Table S2). Among them, the highest frequency was observed for the codon CGU, which encodes arginine (R). Furthermore, we also detected a usage preference of 1 for the codon UGG encoding tryptophan (W) and the codon AUG encoding methionine (M) (Table S2).

**Figure 4:**
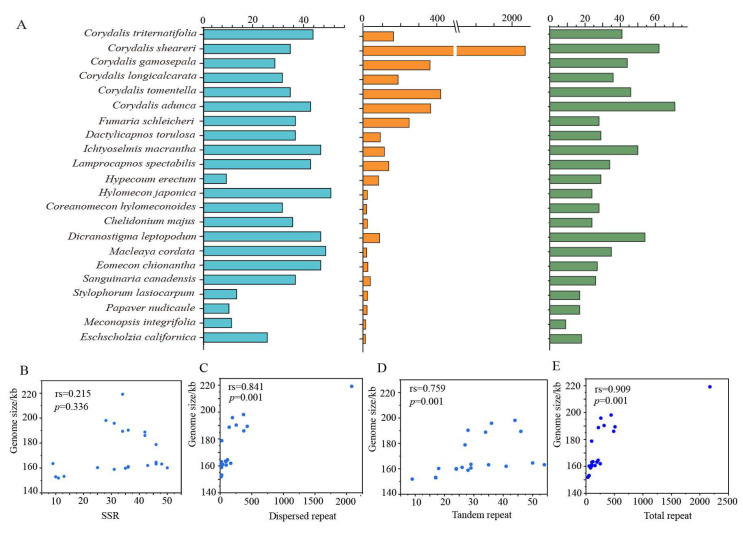
The histogram indicates the number of repetitive sequences ((**A**) left, SSR; middle, dispersed repeat; right, tandem repetitive), and the scatter plot represents the correlation between repetitive sequence numbers and genome size (**B**–**E**).

We detected a total of 732 SSRs, including 678 mononucleotide repeats, 45 dinucleotide repeats, 7 trinucleotide repeats, and 2 hexanucleotide repeats (Figure 4, Table S3). No tetranucleotide repeats and pentanucleotide repeats were detected. The majority of mononucleotide repeats consisted of A/T (96.3%), with C/G accounting for only 3.7%. In addition, we discovered 2999 forward repeats, 1610 palindromic repeats, 28 reverse repeats, and 9 complementary repeats (Figure 4, Table S3). Among them, the maximum number of forward repeats and palindromic repeats in *Corydalis sheareri* was 1079 and 1001, respectively. Complementary repeats were only detected in the *C. adunca*, *Dicranostigma leptopodum*, and *Coreanomecon hylomeconoides* (Figure 4, Table S3). We also detected 749 tandem repeat sequences. Overall, the subfamily of Fumarioideae harbored more tandem repeat sequences compared to the subfamily of Papaveroideae (Figure 4). We tested the correlation between the genome size and the total number of repeats, total tandem repeat number, total SSR number, and total dispersed repeat number, respectively. We found that there is a weak correlation between the genome size and total SSR number (rs = 0.215, *p* = 0.336) (Figure 4B), while the total dispersed repeat number, total tandem repeat number, and total repeat number showed a very strong correlation with the plastid genome size (Figure 4C–E). We also analyzed the correlation between the genome size and total SSR size, total tandem repeat size, and total dispersed repeat size, and similar results were obtained (Figure S3A–C). Furthermore, the GC content was significantly correlated with the size of the repeated sequences (Figure S2D).

### 2.5. Nucleotide Diversity and Positive Selection Analyses

**Figure 5:**
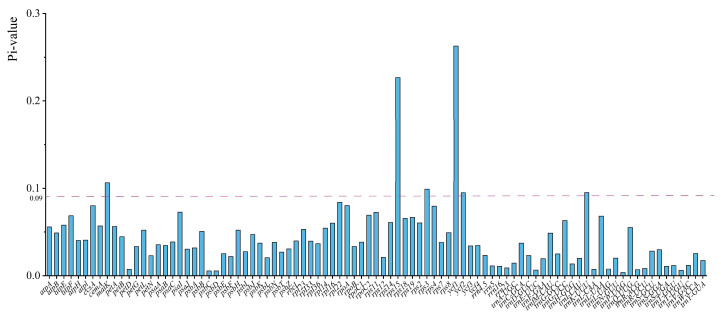
Nucleotide diversity (Pi) of 91 common plastid genes in Papaveraceae.

**Figure 6:**
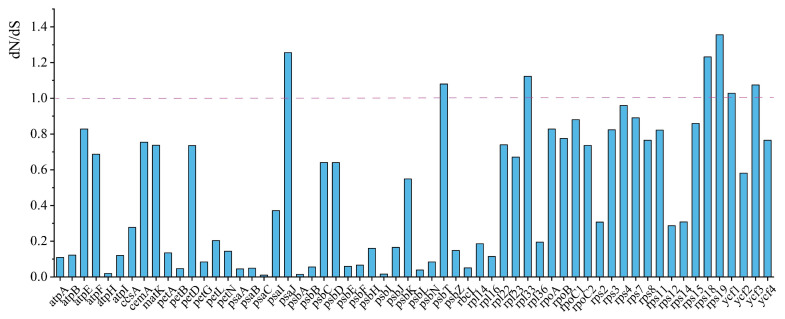
The dN/dS ratio of PCGs in Papaveraceae.

The nucleotide diversity (PI) values were calculated to assess the genetic variation among species within Papaveraceae. The nucleotide diversity for 91 shared genes varied from 0.00357 (*trnP-UGG*) to 0.2627 (*ycf1*), with an average of 0.044 (Figure 5). Apart from *ycf1*, *rps15* (0.2267), *matK* (0.10616), *rps3* (0.09903), *trnK-UUU* (0.09514), and *ycf2* (0.0948) also exhibited high PI values in Papaveraceae (Figure 5). In addition, *ycf1* (0.22656), *ycf2* (0.09397), and *rps3* (0.09206) showed higher pi values in the Hypecooideae and Fumarioideae, while *rps15* (0.19499) and *ycf1* (0.16229) showed higher pi values in the Papaveroideae, indicating their potential suitability as candidate barcodes for the identification of Papaveraceae species in future endeavors (Figure 5 and Figure S3). The ratios of the non-synonymous (dN)/synonymous substitution (dS) rate for the 60 shared PCGs were calculated by the PAML program. We found that the dN/dS values of seven genes (*psaJ*, *psbT*, *rps18*, *rpl33*, *rps19*, *ycf1*, *ycf3*) were significantly greater than 1, indicating positive selection for these genes within Papaveraceae (Figure 6). However, the genes under positive selection showed slight differences among different subfamilies, with *rpl23*, *ycf3*, *rpl33*, and *rps19* in Papaveroideae, and *ycf3*, *rps19*, *rpl33*, *rps18*, and *psaJ* in Fumarioideae and Hypecooideaeare (Figure S4). In addition, most *rps* genes exhibited relatively high dN/dS values, with the majority being above 0.5.

## 3. Discussion

In Papaveraceae, although a large number of plastomes were released, extreme reconfigurable plastomes were only reported in *Corydalis*, *Lamprocapnos,* and *Fumaria* [38,39,40,41], three genera of Fumarioideae. Based on the dense sampling and comprehensive comparison, our study provided a valuable opportunity to further investigate the plastome variation in Papaveraceae. We identified more local collinear blocks showing rearrangements and more genes undergoing loss, pseudogenization, or being truncated in more lineages of Fumarioideae, such as *Dactylicapnos*. Notably, we firstly reported the plastome reconfiguration of Hypecooideae and Papaveroideae. We hypothesized that Papaveroideae plastomes were relatively conserved with the exception of *Eomecon chionantha*, while Fumarioideae and Hypecooideae usually harbored extreme reconfigurable plastomes, which demonstrated a high level of variability in the genome size, gene content, gene order, and rearrangements (Table 1; Figure 2 and Figure 3).

The largest (*Corydalis sheareri*, 219,144 bp) and smallest (*Meconopsis integrifolia*, 151,864 bp) plastome sizes differed significantly (~67 kb, Table S1) in Papaveraceae. In our results, we hypothesized that the genome size variation was mainly due to the IR expansion and the large number of repeat sequences. Moreover, we found that the high variability of the genome size was likely triggered by different factors in different lineages. The plastomes of *Corydalis*, *Fumaria*, *Lamprocapnos,* and *Eomecon* experienced an extreme expansion of their IR region into the SSC region, resulting in one very small SSC region (less than 10 kb) and two very large IR regions (approximately 38~62 kb), which further led to a substantial increase in the total genome size (Figure 3, Table 1). Additionally, more dispersed repeats were also detected in *Corydalis*, *Fumaria,* and *Lamprocapnos* (Figure 4A), which indicated that both the expansion of IRs and large numbers of repeats contributed to the increase in the genome size in these taxa. However, for *Dactylicapnos* and *Ichtyoselmis*, which exhibited a typical IR region (26–27 kb) and a larger SSC region with more than 22 kb (Table 1), a large number of repeats were detected in the SSC region (Table S4), indicating that their slightly larger plastomes were likely caused by the increase of repetitive sequences.

Apart from the variation in the genome size, the expansion of the IR region also significantly contributed to the gene content variation in Papaveraceae (Figure 1 and Figure 3, Table 1). Complete gene duplications (*rpl32*, *trnL-UAG*, *ccsA*, *psaC*, *rps15,* and *ycf1*) were documented due to the expansion of the IR region. In addition, gene loss or pseudogenization occurred frequently for *ndhs*, *accD*, *clpP*, *infA*, *rpl2*, *rpl20*, *rpl32,* and *rps16*, which also resulted in the variation in the gene content. The loss or pseudogenization of the *ndhC*, *ndhJ,* and *ndhK* genes was strongly correlated with the adjacent rearrangement of the LSC region, while the loss or pseudogenization of the remaining *ndh* genes might be related to the IR border shift (Figure 2 and Figure 3), which was similar with previous studies [38,43]. Our results suggested that the loss of the *ndh* genes mainly occurred in two species of *Corydalis*, which belong to different subgenus [50,51], suggesting that there were at least two independent gene loss/pseudogenization events in *Corydalis*. The *ndh* genes encoded subunits of the chloroplast NAD(P)H dehydrogenase (NDH) complex, which is involved in photosystem I (PSI) cyclic electron transfer and chlororespiration [52]. Wicke et al. [53] mentioned that *ndh* gene losses mainly occurred in some groups with a certain degree of heterotrophy, which might render the plastid-encoded *ndh*1 subunit dispensable, a phenomenon not commonly observed in seed plants. However, in recent years, an increasing number of autotrophic species had been reported to have lost the *ndh* genes, such as *Erodium* [54], *Paphiopedilum* [55], and *Cycas* [56]. The *ndh* genes had been suggested as being strongly related to the IR/SSC junction stability [57]. The IR boundary of Papaveraceae was very different from the typical angiosperm boundary, which exhibited high diversity, particularly in *Corydalis*, the largest and most diverse genera of Papaveraceae. We inferred that the loss of the *ndh* genes was likely associated with the IR boundary stability in the poppy family. The *accD* gene encoded acetyl-CoA carboxylase, an enzyme that played a critical role in plants, bacteria, and some eukaryotes [58], and the *clpP* gene encoded a protease that participated in regulating plant growth, development, photosynthesis, and responses to environmental stress [59,60,61]. In our results, the *accD* gene loss was observed in *Corydalis*, *Fumaria,* and *Dactylicapnos*, and the *clpP* gene was lost in *Dactylicapnos* and *Eomecon*. We inferred that the loss of the *accD* gene possibly occurred in the ancestors of *Corydalis*, *Fumaria*, and *Dactylicapnos*, while the *clpP* gene loss occurred multiple times in Papaveraceae.

Coincidentally, the species that lost the *accD* gene, such as *Corydalis*, *Fumaria*, and *Dactylicapnos*, all exhibited extensive rearrangement (Figure 2 and Figure 3). Moreover, one species from Papaveroideae (*Eomecon chionantha*) and one species from Fumarioideae (*Dactylicapnos torulosa*) lost the *clpP* gene. Of those, *Eomecon chionantha* was the only species that exhibited significant IR expansion and rearrangement in Papaveroideae. Additionally, *clpP* was observed as a pseudogene in *Hypecoum erectum*, one species from Hypecooideae, which also exhibited extensive rearrangement. The higher substitution rates in the *accD* and *clpP* genes were correlated with the structural variation in *Hypericum* [62], *Fagopyrum* [63,64], *Oenothera* [65], and Caprifoliaceae [66]. Given all the above evidence, we speculated that the loss of the *accD* and *clpP* genes might be related to plastome rearrangements in Papaveraceae. Moreover, the repetitive sequences in Fumarioideae and Hypecooideae were generally more abundant than those in Papaveroideae (Figure 4B), indicating that the recombination and instability of these repetitive sequences might also contribute to the plastome reconfiguration in Papaveraceae. In addition, the average GC content of Fumarioideae was slightly higher than that in Papaveroideae and Hypecooideae, particularly in *Corydalis*, and the GC content exceeded 40% for most species (Figure 1). In Papaveraceae, the GC content was strongly correlated with the size of the repeated sequences (Figure S2D), which indicated that the variation in the GC content might result from the extreme genome reorganization.

## 4. Materials and Methods

### 4.1. Plant Materials, Taxon Sampling, DNA Extraction, and Sequencing

A total of twenty-two species were sampled, spanning seventeen genera from three subfamilies (Fumarioideae, Hypecooideae, and Papaveroideae) of Papaveraceae. Pteridophylloideae was not sampled due to the fact that it was narrowly distributed in certain regions of Japan. For the two species-rich subfamilies (Fumarioideae and Papaveroideae), five of six previously recognized tribes [26] were collected. For the largest genus, *Corydalis*, more than one species was sampled due to the extreme complexity of the structure variation in previous studies [39,40,41]. The six sampled *Corydalis* species covered all three subgenera and six major clades that were previously recognized [50]. Although a large number of plastomes were reported in previous studies [38,39,40,41,42,50], to eliminate the potential assembly result deviation induced by diverse sequencing methods or software employed by different scholars, we independently sequenced and assembled for a portion of species. In total, thirteen species were newly sequenced, while another ten species, including one outgroup (*Euptelea pleiosperma*), were directly downloaded from the GenBank database (https://www.ncbi.nlm.nih.gov/, accessed on 1 December 2023) or retrieved from our previous studies [51] (Table S1). Total DNA was extracted from silica gel-dried leaves using the modified CTAB (cetyltrimethylammonium bromide) method [67]. The library was constructed with an insert size of approximately 350 bp fragment using the Mgieasy DNA library preparation kit (Beijing Genomics Institution, Shenzhen, China) by following the manufacturer’s instructions. Sequencing was carried out on the BGISEQ-500 platform at BGI, generating 150 bp paired-end (PE) reads. All the raw data have been submitted to the SRA database, and the accession numbers are provided in Table S1.

### 4.2. Plastome Assembly, Annotation, and Plastid Gene Extraction

Clean data were obtained by using SOAPnuke [68] to remove the adapters and low-quality reads with the default parameters. The data quality was assessed using FastQC v0.12.1 [69]. Next, filtered clean reads were assembled de novo using GetOrganelle v1.7.5 [70], and then Bandage v0.8.1 [71] was used to adjust the assembled graphs. The assembled plastome was annotated using PGA [72], with the plastome of *Amborella trichopoda* (AJ506156) as reference. The start/stop codons and intron/exon boundaries of genes were manually modified based on the reference sequences using Geneious Prime v2023.2 (https://www.geneious.com/features/#sequence-analysis, accessed on 1 December 2023). The PCGs, rRNA genes, and tRNAs were separately extracted from the annotated plastome with Geneious Prime. Each gene was aligned using MAFFT v7.450 [73].

### 4.3. Phylogenetic Analyses

Maximum likelihood (ML) analysis was conducted based on 98 concatenated plastid genes with IQ-TREE v1.6.12 [74]. ModelFinder [75] was used to select the best-fit model, and 1000 replications with standard bootstrap support values were performed. Then, the generated tree was visualized and manually improved with FigTree v1.4.4 (http://tree.bio.ed.ac.uk/software/figtree, accessed on 1 December 2023).

### 4.4. Genome Structure Analyses

To determine the potential genomic rearrangements and locally collinear blocks (LCBs), the “progressiveMauve” algorithm implemented in Mauve v2.4.0 [76] was used for comparison, with the plastome of *Euptelea pleiosperma* (NC029429) as reference. CPJSdraw v1.0.0 [77] was employed to assess the expansion and contraction of the IR regions.

### 4.5. Codon Usage and Repeat Sequence Analysis

Due to the degeneracy of codons, most amino acids can be encoded by multiple synonymous codons. The usage frequencies of different codons for different amino acids may not necessarily be the same. Synonymous codon usage bias (SCUB) is species-specific and varies within or among genomes [78]. The utilization of a specific synonymous codon is quantified as the numerator, while the anticipated frequency of that codon’s occurrence serves as the denominator, referred to as the relative synonymous codon usage (RSCU), and it serves as a standard measure of preference. We selected 60 shared PCGs and conducted nucleotide composition analysis using the CodonW v1.4.2 (https://sourceforge.net/projects/codonw/, accessed on 1 December 2023). The identification of simple sequence repeat (SSR) was conducted through the utilization of MISA v2.0 [79], with the minimum number of repeats, mononucleotide, dinucleotide, trinucleotide, tetranucleotide, pentanucleotide, and hexanucleotide repeats were set to 10, 6, 5, 5, 5, and 5, respectively. Tandem repeats sequences were detected using the Tandem Repeats Finder v4.09 [80]. The alignment parameters match, mismatch, delta, match probability, indel probability, minimum alignment score, and maximum period size were set to 2, 7, 7, 10, 50, 80, and 500, respectively. The REPuter [81] was used to detect the dispersed repeats in forward, reverse, complement, and palindromic sequences, with a minimum repeat size set at 30 and a Hamming distance of 3. To determine the correlation of the genome size and the repeat sequence, spearman correlation was performed by SPSS v27.0 [82] under the default settings, and the strength of the correlation was adopted as follows: negligible or very weak (0.1–0.19), weak (0.20–0.29), moderate (0.30–0.39), strong (0.4–0.69), very strong (0.70–0.99), and perfect (1.0) [83].

### 4.6. Nucleotide Diversity and Positive Selection Analyses

The nucleotide diversity of each gene was calculated using DNasp v6.0 [84]. PAML v4.9 [85] was used to calculate the non-synonymous mutation rate (dN) and synonymous mutation rate (dS) of the coding DNA sequences (CDS) under Model 0. The dN/dS > 1, dN/dS = 1, and dN/dS < 1 suggest positive selection, neutral selection, and purifying selection, respectively.

## Figures and Tables

**Table 1:** Characteristics of Papaveraceae plastomes.

Species	Subfamily	Genome Size (bp)	LSC (bp)	IR (bp)	SSC (bp)	GC (bp)	PCG	tRNA	rRNA
*Corydalis triternatifolia*	Fumarioideae	161,946	84,522	38,486	452	38.6	85	37	8
*Corydalis sheareri*	Fumarioideae	219,144	94,030	62,384	346	40.4	99	44	8
*Corydalis gamosepala*	Fumarioideae	198,140	92,169	52,590	791	40.5	95	40	8
*Corydalis longicalcarata*	Fumarioideae	195,842	86,954	54,197	494	40.3	96	38	8
*Corydalis tomentella*	Fumarioideae	189,383	95,825	41,947	9664	40.2	94	38	8
*Corydalis adunca*	Fumarioideae	186,049	100,365	38,100	9484	41.1	92	36	8
*Fumaria schleicheri*	Fumarioideae	190,324	86,789	48,833	5869	40.3	97	40	8
*Dactylicapnos torulosa*	Fumarioideae	160,598	76,421	26,238	31,701	40.3	96	40	8
*Ichtyoselmis macrantha*	Fumarioideae	164,552	88,457	27,031	22,033	39.9	89	37	8
*Lamprocapnos spectabilis*	Fumarioideae	188,795	84,398	51,376	1645	39.2	100	42	8
*Hypecoum erectum*	Hypecoideae	163,471	93,182	27,885	14,519	38.2	86	38	8
*Hylomecon japonica*	Papaveroideae	160,071	88,293	26,712	18,354	38.8	88	37	8
*Coreanomecon hylomeconoides*	Papaveroideae	158,824	86,914	26,686	18,538	38.7	89	37	8
*Chelidonium majus*	Papaveroideae	159,734	87,696	26,735	18,568	38.7	89	37	8
*Dicranostigma leptopodum*	Papaveroideae	163,173	87,793	28,309	18,762	39.4	91	37	8
*Macleaya cordata*	Papaveroideae	163,107	87,919	28,303	18,582	38.6	91	37	8
*Eomecon chionantha*	Papaveroideae	178,768	88,302	45,165	136	37.9	99	38	8
*Sanguinaria canadensis*	Papaveroideae	161,083	87,980	27,372	18,359	38.5	87	37	8
*Stylophorum lasiocarpum*	Papaveroideae	153,196	83,230	25,789	18,388	38.9	89	37	8
*Papaver nudicaule*	Papaveroideae	152,867	83,104	25,731	18,301	38.9	85	37	8
*Meconopsis integrifolia*	Papaveroideae	151,864	82,809	25,653	17,749	38.8	87	37	8
*Eschscholzia californica*	Papaveroideae	160,201	88,147	26,781	18,492	38.7	88	37	8

## Data Availability

All analyzed data for this study are included in the contents of this article and Supplementary Materials.

## Supplementary Materials

The supporting information can be downloaded at: https://www.mdpi.com/article/10.3390/ijms25042278/s1.
