# Supplementary material for: Extreme Reconfiguration of Plastid Genomes in Papaveraceae: Rearrangements, Gene Loss, Pseudogenization, IR Expansion, and Repeats
Source: Int J Mol Sci. 2024 Feb 14;25(4):2278. doi: 10.3390/ijms25042278 (PMC10888665; doi:10.3390/ijms25042278)
Supplement: Supplementary file 1 [file ijms-25-02278-s001.zip › ijms-2839397-supplementary.pdf]

Supplementary Materials

Extreme Reconfiguration of Plastid Genomes in Papaveraceae: Rearrangements, Gene Loss, Pseudogenization, IR Expansion, and Repeats

Jialiang Cao [1], Hongwei Wang [1], Yanan Cao [1], Shenglong Kan [2], Jiamei Li [3,*] and Yanyan Liu [1,*]

[1] College of Plant Protection, Henan Agricultural University, Zhengzhou 450002, China;
caojlcao@163.com (J.C.); whwcas@163.com (H.W.); caoyn47@163.com (Y.C.)
[2] Marine College, Shandong University, Weihai 264209, China;
kanshenglong@sdu.edu.cn
[3] College of Life Sciences, Henan Agricultural University, Zhengzhou 450046, China
* Correspondence: jiamei_li@126.com (J.L.); liuyanyan@henau.edu.cn (Y.L.)

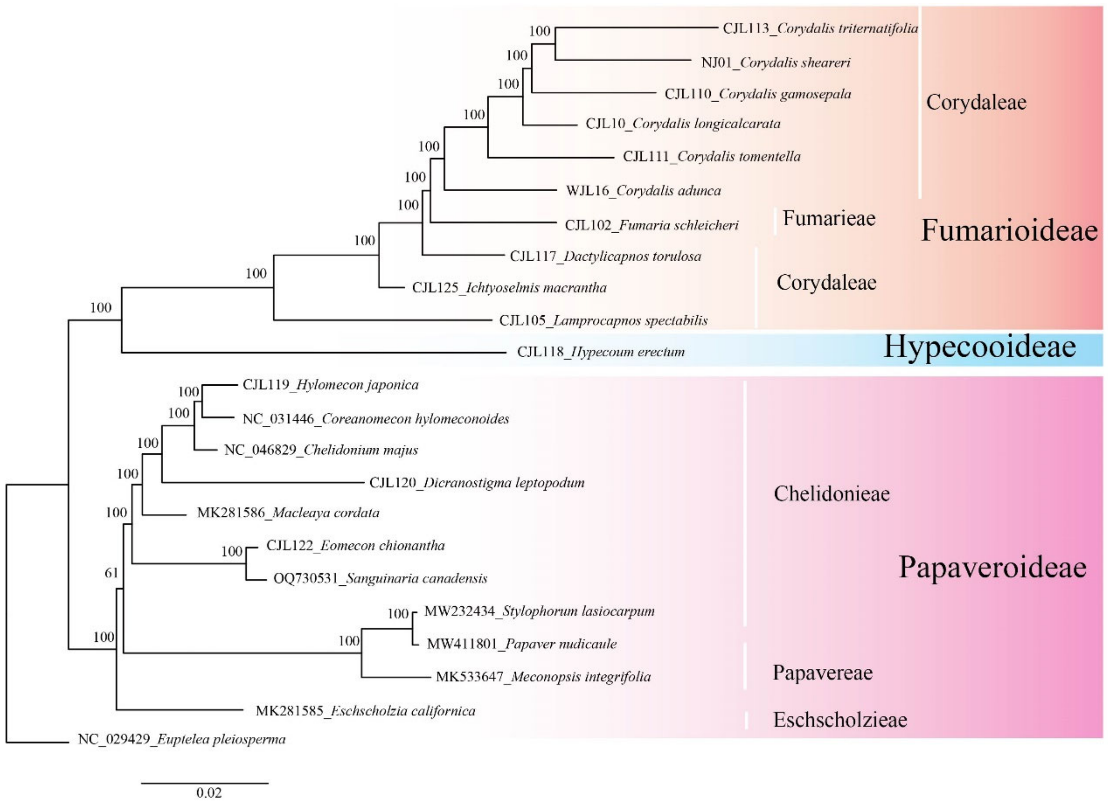

Figure S1. The maximum likelihood (ML) tree constructed based on the 91 shared plastid genes.

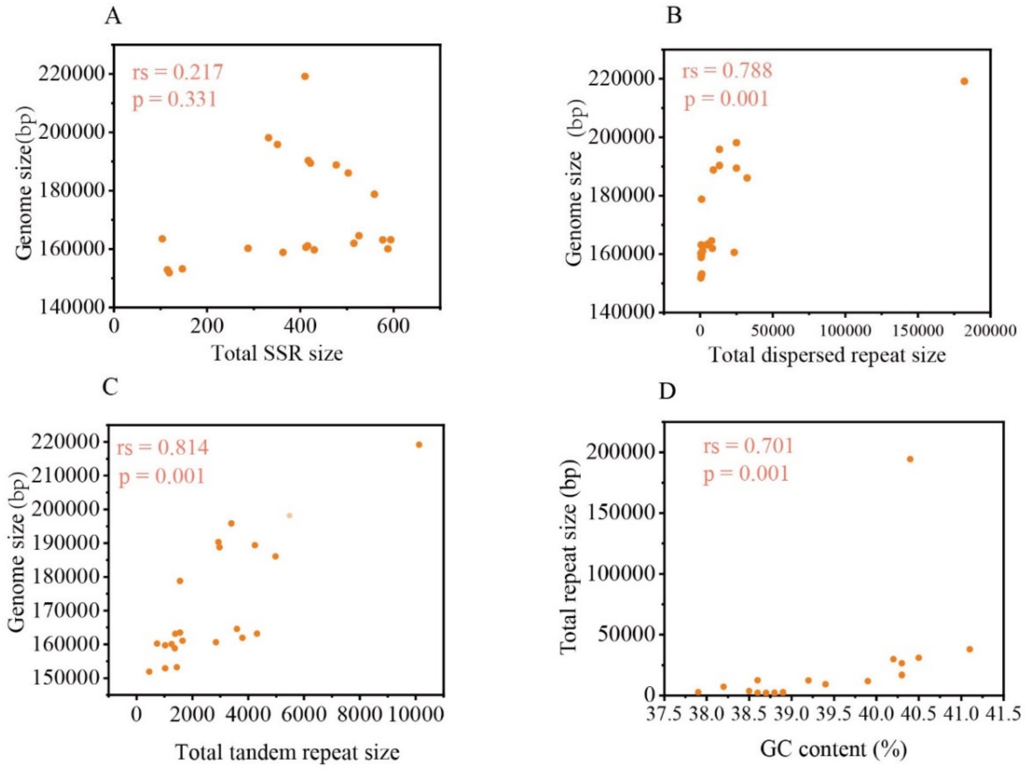

Figure S2. The scatter plots represent the correlation between repetitive sequence numbers or GC content and genome size. A, simple repetitive and genome size; B, dispersed repetitive and genome size; C, tandem repetitive and genome size; D, GC content and total repeat size.

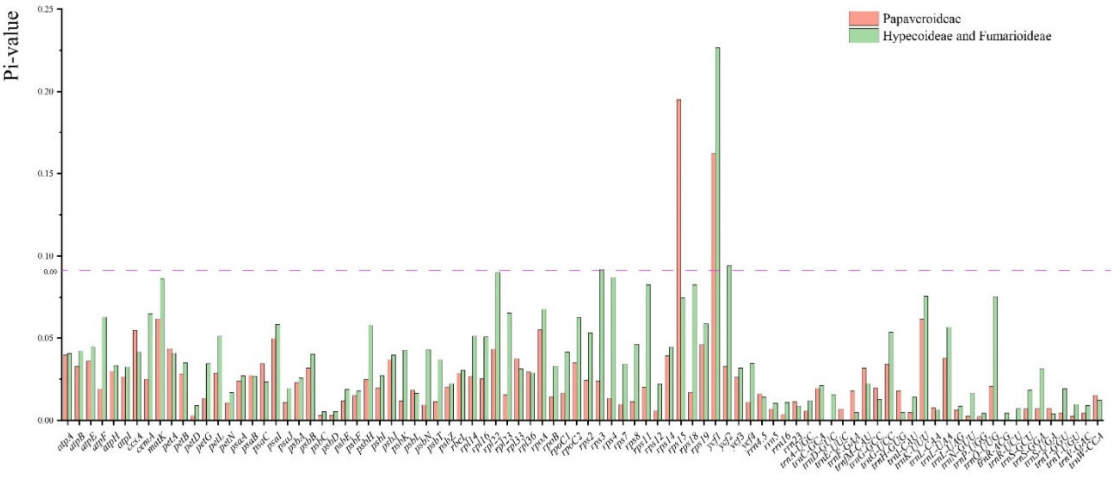

Figure S3. Nucleotide diversity (Pi) of 91 common plastid genes in Papaveroideae, Fumarioideae and Hypecooideae.

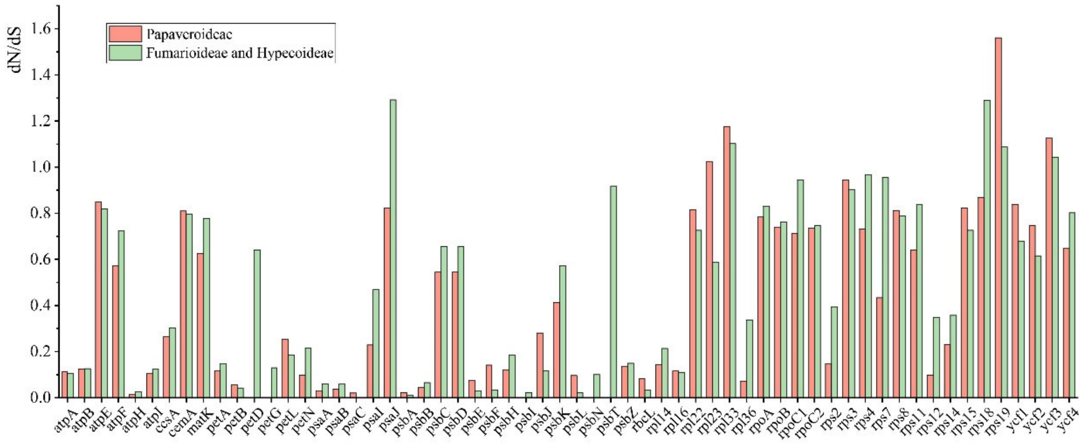

Figure S4. The dN/dS ratio of protein-coding genes in Papaveroideae, Fumarioideae and Hypecooideae.

Table S1. Sampling and genome skimming data characteristics of Papaveraceae in this study.

No.	Species	GenBank accession	Raw reads	Clean reads	Locatity
1	*Corydalis triternatifolia*	SRR26004280	64392108	61612310	Eshan, Yuxi, Yunnan, China
2	*Corydalis sheareri*	-	-	-	Nanjing Botanical Garden Mem. Sun Yat-Sen, Jiangsu, China
3	*Corydalis gamosepala*	SRR26004276	56829644	53806148	Luanchuan, Luoyang, Henan, China
4	*Corydalis longicalcarata*	SRR26004302	52094318	47963554	Wawu Mountain, Hongya, Sichuan, China
5	*Corydalis tomentella*	SRR26004278	60525176	58608832	Fangxian, Shiyan, Hubei, China
6	*Corydalis adunca*	SRR26004251	60891910	45533748	Diebu, Gansu, China
7	*Fumaria schleicheri*	SRR26004262	63635792	59785494	Xiamen University，Fujian，China
8	*Dactylicapnos torulosa*	SRR26004264	57903548	55021766	Kunming, Yunnan,China
9	*Ichtyoselmis macrantha*	SRR26004267	63105304	60520970	Emei Mountain，Sichuan，China
10	*Lamprocapnos spectabilis*	SRR26004263	71700474	64460150	Tianshui，Gansu，China
11	*Hypecoum erectum*	SRR26004265	74356878	64257198	Xinzheng, Henan，China
12	*Hylomecon japonica*	SRR26004268	84697804	64461792	Taibai，Shaanxi，China
13	*Coreanomecon hylomeconoides*	NC_031446	-	-	-
14	*Chelidonium majus*	NC_046829	-	-	-
15	*Dicranostigma leptopodum*	SRR26004269	73774214	64511492	Diebu，Gansu，China
16	*Macleaya cordata*	MK281586	-	-	-
17	*Eomecon chionantha*	SRR26004270	71231612	64307068	Emei Mountain，Sichuan，China
18	*Sanguinaria canadensis*	OQ730531	-	-	-
19	*Stylophorum lasiocarpum*	MW232434	-	-	-
20	*Papaver nudicaule*	MW411801	-	-	-
21	*Meconopsis integrifolia*	MK533647	-	-	-
22	*Eschscholzia californica*	MK281585	-	-	-
23	*Euptelea pleiosperma*	NC029429	-	-	-

Table S2. Codon usage bias in Papaveraceae plastomes.

		RSCU																						
Codon	AA	*C. adunca*	*C. gamosepala*	*C. hylomeconoides*	*C. longicalcarata*	*C. majus*	*C. sheareri*	*C. tomentella*	*C. triternatifolia*	*D. leptopodum*	*D. torulosa*	*E. californica*	*E. chionantha*	*E. pleiosperma*	*F. schleicheri*	*H. erectum*	*H. japonica*	*I. macrantha*	*L. spectabilis*	*M. cordata*	*M. integrifolia*	*P. nudicaule*	*S. canadensis*	*S. lasiocarpum*
GCU	A	0.93	0.95	0.98	0.92	1	0.94	0.97	0.93	1	0.97	1.05	1.01	1.01	0.97	1.03	1	0.97	0.94	1.04	0.99	0.99	1.01	1
GCC	A	0.31	0.31	0.32	0.33	0.31	0.27	0.32	0.32	0.31	0.32	0.32	0.31	0.3	0.29	0.31	0.31	0.32	0.31	0.3	0.34	0.35	0.32	0.34
GCA	A	0.57	0.58	0.61	0.6	0.6	0.58	0.56	0.55	0.6	0.61	0.61	0.62	0.6	0.6	0.68	0.6	0.61	0.57	0.61	0.58	0.62	0.61	0.62
GCG	A	2.19	2.17	2.1	2.15	2.08	2.2	2.15	2.2	2.08	2.1	2.02	2.06	2.09	2.14	1.98	2.08	2.1	2.18	2.05	2.08	2.04	2.06	2.04
UGU	C	0.47	0.5	0.46	0.48	0.46	0.51	0.5	0.52	0.47	0.52	0.48	0.49	0.45	0.49	0.56	0.47	0.46	0.48	0.47	0.44	0.45	0.5	0.45
UGC	C	1.53	1.5	1.54	1.52	1.54	1.49	1.5	1.48	1.53	1.48	1.52	1.51	1.55	1.51	1.44	1.53	1.54	1.52	1.53	1.56	1.55	1.5	1.55
GAU	D	1.82	1.83	1.85	1.84	1.85	1.84	1.83	1.84	1.84	1.81	1.85	1.85	1.85	1.84	1.78	1.84	1.83	1.85	1.85	1.82	1.83	1.85	1.83
GAC	D	0.18	0.17	0.15	0.16	0.15	0.16	0.17	0.16	0.16	0.19	0.15	0.15	0.15	0.16	0.22	0.16	0.17	0.15	0.15	0.18	0.17	0.15	0.17
GAA	E	1.83	1.85	1.85	1.84	1.84	1.84	1.84	1.83	1.85	1.82	1.83	1.85	1.84	1.84	1.81	1.85	1.84	1.84	1.85	1.85	1.84	1.85	1.85
GAG	E	0.17	0.15	0.15	0.16	0.16	0.16	0.16	0.17	0.15	0.18	0.17	0.15	0.16	0.16	0.19	0.15	0.16	0.16	0.15	0.15	0.16	0.15	0.15
UUU	F	1.72	1.72	1.7	1.72	1.7	1.72	1.72	1.72	1.7	1.69	1.7	1.71	1.7	1.72	1.67	1.7	1.71	1.71	1.7	1.73	1.71	1.71	1.71
UUC	F	0.28	0.28	0.3	0.28	0.3	0.28	0.28	0.28	0.3	0.31	0.3	0.29	0.3	0.28	0.33	0.3	0.29	0.29	0.3	0.27	0.29	0.29	0.29
GGU	G	0.63	0.64	0.69	0.63	0.7	0.64	0.64	0.62	0.71	0.65	0.69	0.71	0.7	0.63	0.73	0.71	0.65	0.64	0.7	0.71	0.73	0.7	0.73
GGC	G	2.48	2.45	2.43	2.43	2.42	2.42	2.46	2.45	2.4	2.41	2.41	2.41	2.42	2.45	2.28	2.4	2.44	2.47	2.4	2.41	2.41	2.4	2.42
GGA	G	0.58	0.59	0.58	0.58	0.59	0.59	0.58	0.57	0.59	0.59	0.61	0.6	0.61	0.58	0.65	0.59	0.58	0.57	0.61	0.55	0.57	0.6	0.57
GGG	G	0.32	0.33	0.3	0.35	0.3	0.35	0.32	0.36	0.3	0.35	0.3	0.29	0.27	0.34	0.34	0.3	0.32	0.33	0.29	0.32	0.29	0.3	0.29
CAU	H	1.78	1.79	1.82	1.79	1.82	1.78	1.79	1.79	1.83	1.78	1.82	1.81	1.8	1.79	1.76	1.83	1.79	1.79	1.81	1.8	1.8	1.81	1.8
CAC	H	0.22	0.21	0.18	0.21	0.18	0.22	0.21	0.21	0.17	0.22	0.18	0.19	0.2	0.21	0.24	0.17	0.21	0.21	0.19	0.2	0.2	0.19	0.2
AUU	I	2.48	2.5	2.52	2.49	2.51	2.48	2.5	2.5	2.52	2.44	2.51	2.5	2.51	2.5	2.42	2.52	2.49	2.48	2.5	2.51	2.51	2.5	2.51
AUC	I	0.25	0.22	0.22	0.23	0.21	0.25	0.23	0.24	0.21	0.25	0.22	0.22	0.22	0.24	0.25	0.21	0.24	0.25	0.23	0.22	0.22	0.21	0.22

AUA	I	0.26	0.28	0.26	0.27	0.27	0.27	0.27	0.26	0.27	0.31	0.27	0.28	0.27	0.26	0.33	0.27	0.28	0.27	0.27	0.27	0.27	0.28	0.27
AAA	K	1.89	1.89	1.9	1.89	1.9	1.89	1.9	1.89	1.9	1.88	1.89	1.89	1.89	1.9	1.88	1.9	1.89	1.89	1.89	1.88	1.88	1.89	1.88
AAG	K	0.11	0.11	0.1	0.11	0.1	0.11	0.1	0.11	0.1	0.12	0.11	0.11	0.11	0.1	0.12	0.1	0.11	0.11	0.11	0.12	0.12	0.11	0.12
UUA	L	0.66	0.66	0.73	0.68	0.72	0.69	0.67	0.67	0.72	0.72	0.72	0.73	0.72	0.68	0.85	0.72	0.68	0.72	0.72	0.78	0.78	0.72	0.77
UUG	L	0.43	0.43	0.41	0.41	0.43	0.41	0.42	0.41	0.42	0.43	0.43	0.43	0.42	0.42	0.51	0.42	0.42	0.43	0.43	0.41	0.42	0.42	0.42
CUU	L	0.41	0.42	0.43	0.43	0.42	0.43	0.42	0.44	0.43	0.44	0.42	0.43	0.42	0.44	0.54	0.43	0.43	0.42	0.42	0.47	0.48	0.43	0.47
CUC	L	0.16	0.15	0.13	0.16	0.14	0.16	0.15	0.17	0.14	0.16	0.13	0.14	0.13	0.14	0.12	0.14	0.15	0.14	0.14	0.1	0.12	0.13	0.12
CUA	L	0.28	0.28	0.29	0.28	0.28	0.27	0.29	0.27	0.28	0.28	0.28	0.29	0.28	0.27	0.34	0.28	0.28	0.29	0.28	0.28	0.29	0.29	0.29
CUG	L	4.05	4.06	4	4.05	4.01	4.04	4.05	4.03	4.01	3.96	4.01	3.98	4.02	4.05	3.64	4.01	4.03	4	4.01	3.96	3.9	4	3.93
AUG	M	1	1	1	1	1	1	1	1	1	1	1	1	1	1	1	1	1	1	1	1	1	1	1
AAU	N	0.41	0.41	0.38	0.41	0.38	0.41	0.41	0.43	0.38	0.43	0.39	0.41	0.38	0.39	0.48	0.38	0.39	0.39	0.38	0.38	0.39	0.4	0.38
AAC	N	1.59	1.59	1.62	1.59	1.62	1.59	1.59	1.57	1.62	1.57	1.61	1.59	1.62	1.61	1.52	1.62	1.61	1.61	1.62	1.62	1.61	1.6	1.62
CCU	P	0.61	0.59	0.65	0.59	0.64	0.6	0.6	0.56	0.66	0.63	0.66	0.63	0.64	0.62	0.78	0.66	0.62	0.59	0.66	0.65	0.68	0.62	0.67
CCC	P	0.28	0.26	0.3	0.26	0.32	0.26	0.27	0.29	0.3	0.28	0.29	0.33	0.31	0.29	0.3	0.3	0.27	0.27	0.3	0.28	0.28	0.34	0.28
CCA	P	0.38	0.38	0.39	0.38	0.39	0.38	0.38	0.39	0.4	0.39	0.43	0.4	0.39	0.4	0.47	0.4	0.38	0.39	0.41	0.42	0.44	0.41	0.44
CCG	P	2.73	2.77	2.66	2.76	2.65	2.75	2.75	2.76	2.64	2.71	2.62	2.63	2.66	2.7	2.46	2.64	2.73	2.75	2.63	2.65	2.61	2.63	2.61
CAA	Q	0.49	0.5	0.51	0.5	0.5	0.51	0.51	0.52	0.51	0.51	0.52	0.51	0.5	0.52	0.65	0.51	0.51	0.52	0.5	0.55	0.54	0.51	0.54
CAG	Q	1.51	1.5	1.49	1.5	1.5	1.49	1.49	1.48	1.49	1.49	1.48	1.49	1.5	1.48	1.35	1.49	1.49	1.48	1.5	1.45	1.46	1.49	1.46
CGU	R	4.74	4.74	4.71	4.7	4.73	4.71	4.69	4.67	4.73	4.65	4.74	4.76	4.76	4.72	4.59	4.73	4.73	4.73	4.76	4.7	4.67	4.76	4.69
CGC	R	0.13	0.13	0.17	0.15	0.14	0.15	0.16	0.17	0.17	0.15	0.15	0.13	0.14	0.13	0.13	0.17	0.13	0.14	0.14	0.15	0.18	0.13	0.17
CGA	R	0.37	0.39	0.35	0.37	0.36	0.39	0.39	0.39	0.34	0.39	0.32	0.35	0.35	0.39	0.43	0.34	0.36	0.37	0.35	0.34	0.36	0.35	0.35
CGG	R	0.11	0.1	0.1	0.13	0.1	0.11	0.13	0.13	0.1	0.12	0.11	0.09	0.09	0.11	0.09	0.1	0.12	0.11	0.1	0.12	0.13	0.09	0.13
AGA	R	0.49	0.47	0.52	0.49	0.51	0.48	0.48	0.47	0.51	0.52	0.51	0.53	0.53	0.5	0.58	0.51	0.5	0.48	0.51	0.55	0.53	0.53	0.53
AGG	R	0.15	0.16	0.15	0.15	0.15	0.16	0.15	0.17	0.16	0.17	0.17	0.14	0.14	0.15	0.18	0.16	0.15	0.16	0.14	0.15	0.13	0.14	0.13
UCU	S	0.56	0.63	0.57	0.57	0.57	0.57	0.56	0.56	0.58	0.61	0.55	0.56	0.56	0.57	0.71	0.58	0.57	0.57	0.54	0.56	0.57	0.57	0.57
UCC	S	0.33	0.31	0.36	0.33	0.37	0.34	0.35	0.37	0.34	0.34	0.34	0.35	0.36	0.35	0.45	0.34	0.31	0.34	0.37	0.34	0.34	0.35	0.34

UCA	S	0.35	0.35	0.34	0.36	0.34	0.34	0.38	0.33	0.34	0.37	0.38	0.36	0.37	0.35	0.39	0.34	0.36	0.33	0.36	0.4	0.39	0.36	0.39
UCG	S	0.18	0.16	0.16	0.19	0.15	0.17	0.16	0.2	0.17	0.18	0.14	0.16	0.15	0.17	0.17	0.17	0.18	0.17	0.15	0.11	0.12	0.17	0.12
AGU	S	0.37	0.39	0.4	0.39	0.42	0.42	0.39	0.4	0.41	0.41	0.37	0.39	0.38	0.39	0.53	0.41	0.38	0.39	0.41	0.41	0.41	0.4	0.41
AGC	S	4.21	4.17	4.17	4.17	4.15	4.16	4.15	4.14	4.16	4.09	4.22	4.18	4.18	4.17	3.75	4.16	4.2	4.2	4.15	4.18	4.16	4.15	4.17
ACU	T	0.67	0.67	0.73	0.68	0.72	0.69	0.7	0.68	0.72	0.73	0.71	0.75	0.7	0.7	0.86	0.72	0.7	0.7	0.7	0.73	0.74	0.75	0.74
ACC	T	2.73	2.74	2.67	2.72	2.67	2.71	2.72	2.7	2.68	2.65	2.7	2.66	2.74	2.7	2.46	2.68	2.71	2.69	2.71	2.67	2.64	2.66	2.65
ACA	T	0.42	0.42	0.44	0.42	0.43	0.42	0.41	0.42	0.42	0.43	0.41	0.44	0.41	0.4	0.49	0.42	0.42	0.43	0.42	0.44	0.46	0.44	0.45
ACG	T	0.19	0.17	0.16	0.19	0.18	0.18	0.17	0.2	0.17	0.19	0.17	0.15	0.15	0.2	0.18	0.17	0.16	0.18	0.17	0.16	0.16	0.16	0.16
GUU	V	0.58	0.57	0.6	0.6	0.58	0.59	0.57	0.58	0.59	0.58	0.58	0.61	0.59	0.56	0.62	0.59	0.56	0.63	0.59	0.59	0.6	0.6	0.59
GUC	V	0.28	0.28	0.21	0.24	0.21	0.27	0.26	0.3	0.22	0.28	0.22	0.22	0.19	0.24	0.25	0.22	0.26	0.23	0.21	0.23	0.25	0.23	0.25
GUA	V	0.65	0.64	0.74	0.65	0.73	0.64	0.64	0.62	0.74	0.67	0.71	0.71	0.71	0.68	0.82	0.74	0.67	0.68	0.73	0.7	0.71	0.72	0.71
GUG	V	2.5	2.52	2.45	2.51	2.47	2.5	2.53	2.5	2.46	2.47	2.49	2.46	2.51	2.52	2.31	2.46	2.51	2.46	2.48	2.47	2.44	2.46	2.45
UGG	W	1	1	1	1	1	1	1	1	1	1	1	1	1	1	1	1	1	1	1	1	1	1	1
UAU	Y	1.83	1.84	1.85	1.85	1.87	1.86	1.84	1.83	1.86	1.83	1.84	1.86	1.85	1.85	1.82	1.86	1.83	1.85	1.86	1.87	1.87	1.86	1.87
UAC	Y	0.17	0.16	0.15	0.15	0.13	0.14	0.16	0.17	0.14	0.17	0.16	0.14	0.15	0.15	0.18	0.14	0.17	0.15	0.14	0.13	0.13	0.14	0.13

Table S3. Numbers of SSR, tandem repeat, and dispersed repeat in Papaveraceae plastomes.

Species	Simple sequence repeat																	Tandem repeat	Dispersed repeat				Total
	A	C	G	T	AT	TA	AG	TG	CA	CT	CAA	AAT	ATT	ATC	CATCCT	GAGGAT	TCCTCA		F	P	R	C	
Corydalis triternatifolia	25	1	-	14	3	-	-	-	-	-	-	-	-	-	-	-	-	41	160	8			252
Corydalis sheareri	8	1	-	23	1	1	-	-	-	-	-	-	-	-	-	-	-	62	1079	1001	1		2177
Corydalis gamosepala	3	-	1	22	1	-	-	-	-	-	1	-	-	-	-	-	-	44	279	85	6		442
Corydalis longicalcarata	12	-	1	17	-	1	-	-	-	-	-	-	-	-	-	-	-	36	138	57			262
Corydalis tomentella	9	1	-	17	3	3	-	-	-	-	1	-	-	-	-	-	-	46	388	40	2		510
Corydalis adunca	22	1	-	14	3	1	1	-	-	-	-	-	-	-	-	-	-	71	202	154	11	7	487
Fumaria schleicheri	20	1	1	14	-	-	-	-	-	-	-	-	-	-	-	-	-	28	185	70			319
Dactylicapnos torulosa	16	2	1	14	-	1	1	-	-	1	-	-	-	-	-	-	-	29	63	33			161
Ichtyoselmis macrantha	18	1	1	23	1	2	-	-	-	-	-	-	-	-	-	-	-	50	104	14			214
Lamprocapnos spectabilis	15	-	1	23	1	-	-	1	-	-	-	-	-	-	-	1	-	34	122	21			219
Hypecoum erectum	1	-	-	2	3	3	-	-	-	-	-	-	-	-	-	-	-	29	54	29	3		124
Hylomecon japonica	20	1	-	25	1	3	-	-	-	-	-	-	-	-	-	-	-	24	14	10	1		99
Coreanomecon hylomeconoides	11	1	-	17	1	-	-	-	-	-	-	-	1	-	-	-	-	28	11	9	1	1	81
Chelidonium majus	15	3	-	15	-	1	-	-	-	-	-	-	1	-	-	-	-	24	14	10			83
Dicranostigma leptopodum	22	2	1	18	-	3	-	-	-	-	-	-	-	-	-	-	-	54	83	8		1	192
Macleaya cordata	27	1	-	19	-	-	-	-	-	-	-	-	-	-	1	-	-	35	11	10			104
Eomecon chionantha	21	-	1	22	1	-	-	-	-	-	-	-	-	1	-	-	-	27	18	9			100
Sanguinaria canadensis	19	1	-	12	3	-	-	-	-	-	-	-	-	1	-	-	-	26	27	11	3		103

Stylophorum lasiocarpum	6	-	-	7	-	-	-	-	-	-	-	-	-	-	-	-	-	17	17	9	56
Papaver nudicaule	5	-	-	5	-	-	-	-	-	-	-	-	-	-	-	-	-	17	15	8	50
Meconopsis integrifolia	5	-	-	6	-	-	-	-	-	-	-	-	-	-	-	-	-	9	8	7	35
Eschscholzia californica	9	-	-	15	-	-	-	-	-	-	-	1	-	-	-	-	-	18	7	7	57
